# Extracorporeal Selective Chloride Removal By Electrodialysis: An Innovative Treatment For Respiratory and Metabolic Acidosis

**DOI:** 10.1186/2197-425X-3-S1-A502

**Published:** 2015-10-01

**Authors:** A Zanella, L Caironi, P Castagna, M Giani, S Abd El Aziz El Sayed Deab, E Scotti, M Chiodi, F Zadek, S Colombo, D Salerno, L Gattinoni, A Pesenti

**Affiliations:** Università degli Studi di Milano Bicocca, Monza, Italy; Università degli Studi di Milano, Milano, Italy; Ospedale Maggiore Policlinico, Milano, Italy; Fondazione IRCCS Ca' Granda - Ospedale Maggiore Policlinico, Milano, Italy; Ospedale San Gerardo, Monza, Italy

## Introduction

Acidosis is a frequent disorder among critically ill patients. When patient compensatory responses fail to restore a normal pH, administration of sodium bicarbonate (NaHCO_3_) or renal replacement therapy may be required. Intravenous NaHCO_3_ increases plasma Strong Ion Difference ([SID] = [Na^+^] + [K^+^] - [Cl^-^]) and HCO_3_^-^ concentration by raising Na^+^ concentration. Although effective, this treatment is not devoid of complications, such as hypernatremia, hyperosmolarity and fluid overloading[1]. Selective chloride (Cl^-^) removal, by increasing SID in an alternative way, may allow a rapid correction of acidosis without altering plasma osmolality and Na^+^ concentration.

## Objectives

In an experimental animal model of severe respiratory and metabolic acidosis, we aimed to assess the efficacy of an electrodialytic system, able to selectively remove anions from plasma ultrafiltrate, to normalize pH.

## Methods

Seven sedated and paralyzed healthy swine were connected to a veno-venous extracorporeal circuit including a dialyzer and an electrodialysis unit. Animals underwent 2 randomly-ordered experimental sequences of respiratory and metabolic acidosis, obtained by reducing the respiratory rate or by continuous infusion of lactic acid, respectively, targeting an arterial pH of 7.15 ± 0.02. the electrodialysis treatment was then started to restore baseline pH. Hemodynamics, acid-base equilibrium, and laboratory parameters were recorded.

## Results

**Figure 1:**
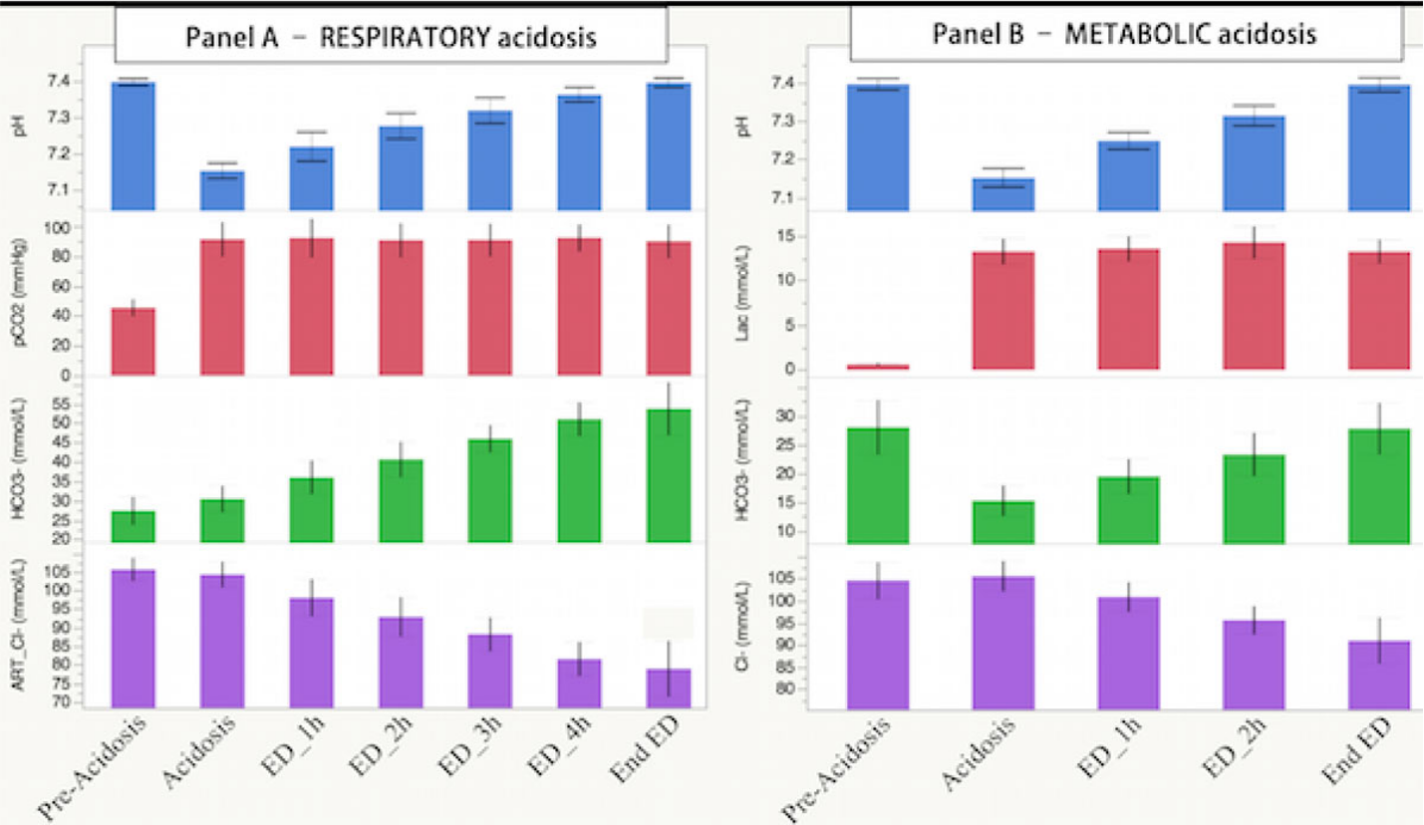
***Chloride removal. Panel A: Arterial pH, pCO***_***2***_ ***, HCO***_***3***_^***-***^ ***, and Cl*** ^***-***^ ***concentration over time during respiratory acidosis. Panel B: Arterial pH, lactate, HCO***_***3***_^***-***^ ***and Cl*** ^***-***^ ***concentration over time during metabolic acidosis. After induction of acidosis, the electrodialysis treatment started.***

An arterial pCO_2_ of 91 ± 11 mmHg and a lactate concentration of 13.2 ± 1.4 mmol/L were required to achieve the targeted respiratory and metabolic acidosis, respectively. the electrodialysis treatment restored the baseline pH by reducing plasma Cl^-^ concentration respectively from 105 ± 4 to 79 ± 8 mEq/L in 306 ± 54 min (for respiratory acidosis), and from 105 ± 3 to 91 ± 5 mEq/L in 175 ± 47 min (for metabolic acidosis) (p < 0.001 for both, see Figure 1). No adverse events ascribable to the treatment were recorded.

## Conclusions

Selective extracorporeal removal of Cl^-^ by electrodialysis is a feasible, rapid and effective in-vivo treatment to completely reverse severe respiratory or metabolic acidosis.
